# Diagnosis and medical treatment of neuropathic pain in leprosy**^1^**

**DOI:** 10.1590/1518-8345.0676.2731

**Published:** 2016-08-08

**Authors:** Rogerio Del Arco, Susilene Maria Tonelli Nardi, Thiago Gasperini Bassi, Vania Del Arco Paschoal

**Affiliations:** 2 MSc, Physician Specialist in Neurosurgery.; 3 PhD, Researcher, Departamento de Pesquisa, Instituto Adolfo Lutz, São José do Rio Preto, SP, Brazil.; 4 Neurologist.; 5 PhD, Adjunct Professor, Faculdade de Medicina de São José do Rio Preto, São Jose do Rio Preto, SP, Brazil.

**Keywords:** Leprosy, Neurologic Manifestations, Disabled Persons, Pain

## Abstract

**Objective:**

to identify the difficulties in diagnosing and treating neuropathic pain caused by leprosy and to understand the main characteristics of this situation.

**Methods:**

85 patients were treated in outpatient units with reference to leprosy and the accompanying pain. We used a questionnaire known as the Douleur Neuropathic 4 test and we conducted detailed neurological exams. As a result, 42 patients were excluded from the study for not having proved their pain.

**Results:**

Out of the 37 patients that experienced pain, 22 (59.5%) had neuropathic pain (or a mixture of this pain and their existing pain) and of these 90.8% considered this pain to be moderate or severe. 81.8% of the sample suffered with this pain for more than 6 months. Only 12 (54.5%) of the patients had been diagnosed with neuropathic pain and in almost half of these cases, this pain had not been diagnosed. With reference to medical treatment (n=12) for neuropathic pain, 5 (41.6%) responded that they became better. For the other 7 (58.4%) there were no changes in relation to the pain or in some cases the pain worsened in comparison to their previous state. Statistical analysis comparing improvements in relation to the pain amongst the patients that were treated (n=12) and those that were not, showed significant differences (value p=0.020).

**Conclusion:**

we noted difficulties in diagnosing neuropathic pain for leprosy in that almost half of the patients that were studied had not had their pain diagnosed. We attributed this to some factors such as the non-adoption of the appropriate protocols which led to inadequate diagnosis and treatment that overlooked the true picture.

## Introduction

The presence of pain is a common characteristic in patients with leprosy. It is responsible for physical and psychological pain(1). The cause of the pain is connected to secondary nociceptive stimulus and tissue inflammation which is often triggers off episodes of immune activation (reverse reaction and erythema nodosoum leprosum). Alternatively there is a neuropathic cause that is secondary to the damage which causes a complete lack of functioning of the nervous system (2).

The International Association for the Study into Pain (IASP) defines the pain as being caused by lesions or somatosensory nervous system diseases. Neuropathic pain is a type of pain that has been ignored in the treatment of those with leprosy (3-4). For a long time it was attributed to inflammatory processes, or even the process of neural compression - nociceptive pain, being responsible for the entire pain for this group of patients. 

Erroneous diagnosis ended up being prejudicial for the patients that were not treated appropriately as well as causing comorbidities such as gastric problems, osteoporosis, neuropathy amongst others. This occurred due to the excessive use of anti-inflammatories particularly corticosteroids. This with analgesics, made up the few tools that health practitioners had. 

**Figure 1:**
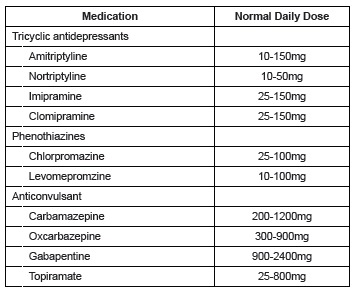
Suggested medication for the treatment of neuropathic pain in Brazil*

The Brazilian Medical Association (AMB) advocates that treatment of neuropathic pain should involve the use of 3 classes of medication namely: tricyclic antidepressants (amitriptyline, nortriptyline, imipramine and clomipramine), Phenothiazines neuroleptics (chlorpromazine, levomepromzine) and anticonvulsants (carbamazepine, gabapentine, oxcarbazepine, topiramate, pregabalin) that can be associated with analgesics and anti-inflammatories according to the needs of every patient (2) (Figure 1).

*Modified by Garbino JA et al. Leprosy: Diagnosis and Treatment of Neuropathy. Directive Project, Brazilian Medical Association and the Federal Council of Medicine 2003; 147–159

In the main specialist journals on neuropathic pain for leprosy, we sought to analyze epidemiological and ethological aspects of this problem, focusing on the prevalences and trying to infer causal relations or evaluating the psychological state of patients (1-5). Different to previous studies, this study concerned itself with identifying the difficulties in diagnosing and treating neuropathic pain caused by leprosy as well as to understand the main characteristics of this situation.

## Method

This was a descriptive and transversal study. We started by collecting data from medical reports and through the application of protocols on 85 patients that were at outpatient units in relation to leprosy in a large municipality in Brazil in 2013. The project was approved by the Ethics Committee at the Medical School in São José in Rio Preto (FAMERP), CAAE 02435120.00005414.

Researchers carried out the research after having explained to the participants the reasons for the study. The subsequently signed a Consent Form (TCLE) indicating that they took part of the own free will. Then a document was drawn up with demographical and epidemiological data on the patients and on the historical development of the disease, particularly related to diagnosis of neuropathic pain. We developed a scale in relation to the pain and an anatomical localization of the pain as well as noting the characteristics of the treatment with special reference to the medication used.

All of the patients included in the study were classified in accordance with the criteria adopted for leprosy by the World Health Organization (WHO) and they were not being treated with Polychemotherapy (PQT) in that they had not received a minimum of 6 doses in Paucibacillary form (PB) and 12 doses in Multibacillary form (MB)^6^ ^-^ ^7^.

At the time of the interview, none of the patients showed signs or symptoms of having a reactionary state such as Reverse Reaction (RR) or Erythema Nodosoum Leprosum (ENH). In order to obtain a diagnosis of neuropathic pain, patients should complaint of pain that has not been caused by stimulus and anatomical plausibility - in one or more of the regions related to the affected nerve^3^ ^,^ ^8^ ^-^ ^9^. 

We opted to use the *Douleur Neuropathic 4 Questionary* (DN4) questionnaire that was translated and checked by a Portuguese person called Santos et al in 2009. This questionnaire was chosen because of its ease of use, its sensitivity (83%) and its precision (90%) in predicting the presence of pain with the characteristics of neuropathy^11^.

After having defined the case as stemming from neuropathy we used the *Numeric Rating Scale -* NRS to understand better the intensity and the area of the pain (the process is known as Pain Drawing - PD)(12-13).

Deformities were evaluated using the WHO criteria: Level zero - no deformity, Level 1 - loss of sensitivity in the hands and feet or Level 2 - visible motor deformity, including lagophthalmos and the gripping of fingers and contractures and/or ulcers on the hands and feet(14).

**Figure 2:**
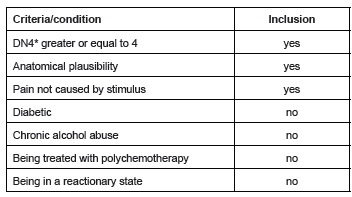
The inclusion criteria used for the patients whose pain stemmed from neuropathy. São José in Rio Preto, SP, 2014

In Figure 2 the inclusion criteria used for the patients whose pain stemmed from neuropathy, can be seen. We opted to exclude patients that were diagnosed with diabetes Mellitus and/or alcoholism so there would be no confusion regarding possible diagnosis of neuropathy and the aforementioned diseases being other possible causes.

*The DouleurNeuropathic 4 Questionary questionnaire

The results were analyzed using the Microsoft Excel statistical program 2013 and Graf Pad Instat 3.00/1997. We checked the significance of the results through the test t for Students, for parametric variables and Mann-Whitney for the non-parametric variables, where appropriate. We considered the limit of statistical significance to be the value of p≤0,05. 

## Results

Out of the 85 patients who had leprosy that we studied, 37 (43.5%) were in a painful nociceptive state and/or were neuropathic and 48 (56.5%) were excluded for not having complained of pain or for just having referred to it in the past.

After having applied the questions to diagnose the cause of the pain to be neuropathic in the 37 patients who complained of pain, we noted that 12 of them did not match up to the inclusion criteria (Figure 2) and were thus excluded. The remaining 22 patients that complained of neuropathic pain or a mixture of other pains represented 25.9% of the total population that was analyzed (n=85) and they were the main focus of this study.

### General Characteristics 

The following are details on the profile of those affected by neuropathic pain related to leprosy in our study: 14 (63.7%) were women and their age varied from 24 to 66 years old, with an average age of 51 (dp±10,78). 

Fifteen (68.1%) patients that were affected showed a form of MB. We noted that for those who had been diagnosed for a long period of time there was a greater frequency of neuropathic pain. 14 (63.7%) had known about the disease for more than 5 years with 11 (50%) having undergone PQT for more than 5 years. The existence or previous existence of a reactionary state did not show that this in itself had any bearing on the neuropathic pain.

The clinical characteristics are shown below in Table 1. 

**Table 1:** General Characteristics of the patients with neuropathic pain. São José in Rio Preto, SP, 2014

General Characteristics of the patients with neuropathic pain (N=22)		N	%
Gender	Male	8	36.3
	Female	14	63.7
Classification of the treatment by the WHO*	Paucibacillary	7	31.9
	Multibacillary	15	68.1
Time of the diagnosis for leprosy	<5 years	8	36.3
	>5 years	14	63.7
Time when the polychemotherapy ends	<1 year	5	22.7
	1 to 5 years	6	27.3
	>5 years	11	50
Previous history of reactionary state	Yes	11	50
	No	11	50
Time of the first symptons	<6 months	4	18.2
	>6 months	18	81.8
Characteristics of the first symptons	Sudden	1	4.6
	Gradual	21	95.4
Intensity of pain - NRS^†^	Lighht (1 to 3)	2	9.1
	Moderate (4 to 6)	15	68.2
	Severe (7 to10)	5	22.7
Pattern of the pain	Superficial	Zero	Zero
	Deep	17	77.3
	Both	5	22.7
Level of incapacity - WHO*	Zero	2	9.2
	Level 1	14	63.6
	Level 2	6	27.2

*World Health Organization

†Numeric Rating Scale

### Diagnosis and Treatment 

Out of the 22 patients included in the study for having neuropathic pain caused by leprosy, 10 (45.5%) were misdiagnosed cases. Dermatologists gave diagnosis in 66.6% of the cases. There were no mistakes in the diagnosis (false-positive) on the part of the medical assistant.All of the diagnosed patients received, at some point, treatment with medication recommended for cases of neuropathic pain. 

Tricyclic antidepressants were the most used class of medication. At one point, all of the 12 cases treated received a prescription for amitriptyline. Two patients stopped using the medication due to somnolence, dry mouth and constipation. One patient stopped the having the treatment believing that the medication for the treatment was for depression.

Two patients failed in obtaining improvements for their pain due to insufficient doses of amitriptyline, being doses below 10 to 25mg per day. The non-usage of polytherapy and the use of doses of, on average, 50mg per day explains why there were no symptoms of improvements.

In the class of medications known as anticonvulsants, carbamazepine was used by 8 patients. It was subsequently substituted by gabapentine in 1 case and by pregabalin in another since the first medication did not produce the desired results. The patients stated that there were improvements after having used pregabalin and gabapentine. 

Another case of treatment failure occurred with a patient that was undergoing polytherapy (with amitriptyline + carbamazepine). The patient stopped the treatment in using carbamazepine complaining of side effects (somnolence and dizziness). The patient did not receive other alternative medication that could have been used to resolve the problem. The phenothiazine class of medication was not received by any of the patients.

None of the 12 patients that were studied (8 of which underwent polytherapy) were given the maximum doses of medication used in the treatment of neuropathic pain. 

Two of the patients obtained their medication through spending their own money because gabapentine and pregabalin is not provided on the Brazilian National Health Service (SUS) (Table 2).

**Table 2:** Characteristics of the diagnosis and treatment of patients with neuropathic pain caused by leprosy. São José in Rio Preto, SP, 2014

Characteristics of the diagnosis and treatment of patients with neuropathic pain	Situation	n	%
Diagnosed for neuropathic pain (n=22)	Yes	12	54.5
	No	10	45.5
Specialist that carried out the diagnosis (n=12)	Dermatologist	8	66.6
	Neurologist	4	33.4
Response to the treatment (n=12)	Improvements in the pain	5	41.6
	Equal or worse	7	58.4
Type of medication used in the treatment (n=12)	Monotherapy	4	33.4
	Polytherapy	8	66.6
Causes for failures in the therapy used (n=7)	Stopped due to side effects	3	42.9
	Unaware of the medication	1	14.3
	Taking inadequate doses	2	28.5
	Lack of polytherapy	1	14.3
The way how the medication was acquired (n=12)	Government	10	83.3
	Government + Private Sector	2	16.7

There were improvements for the patients that were treated in comparison to those that were not with medication. The information was obtained when they were questioned (n=12), (valor de p=0,020). There were no significant differences in relation to the intensity of the pain and the DN4 points between patients that were treated for neuropathic pain (n=12) and those that were not (n=10). (Table 3).

**Table 3:** Comparison between cases that were treated and were not treated for neuropathic pain caused by leprosy. São José in Rio Preto, SP, 2014

Variables	Treated (n=12)	Non treated (n=10)	Value of p
Improvements in the pain	5	0	0.0202*
Intensity of pain - (average)	7.5	6	0.4652^†^
*Points on the Douler Neuropathic 4 Questionary questionnaire (average)	7	6.5	0.0692^†^

*Student Test t; †Mann-Whitney Test

## Discussion

One in four cases of patients treated for leprosy were affected by neuropathic pain, according to the study (25,8%). The symptoms are very common according to studies from China (2012) and India (2011) which showed frequencies of 45.8% and 21.8% respectively(1,15).

This is an important cause of the patients suffering. 90.8% stated that the pain was moderate and/or severe and 81.8% stated that they suffered for longer than six months. Nearly half of the cases studied did not have a diagnosis (45.5%). This finding highlights the fact that neuropathic pain cause by leprosy is not being picked up by health care teams. They spend most of their time looking for new cases of the disease and using polychemotherapy for existing cases. They deal with reactionary episodes and the prevention of deformities as well as the controlling communicants ^4^ ^,^ ^16^.

What could reduce the failures in diagnosing this condition is the adoption of a protocol for the identification of neuropathic pain. The DN4 was used in this study. It was translated into Portuguese and can be used by any trained professional.

Patients symptoms can be minimized where professionals are made aware of the medications used for chronic/neuropathic pain and if the cases are handled appropriately.

The use of medications for treatment of neuropathic pain caused by leprosy has not been fully tested. Random and control tests are needed for class I and II medications which are not currently covered by medical journals. A good understanding of these therapies can alleviate symptoms and prevent neuropathic lesions for patients with leprosy^16^ ^-^ ^18^. A revision of the studies on treatment of various causes of neuropathic pain has included, in the majority of cases, ill people with severe polyneuropathy diabetes and postherpetic neuralgia ^19^.

Medication treatment for neuropathic pain (as recommended by AMB) ought to commence with the application of low doses and then it can be increased on a gradual basis where the pain persists. The herpetic and renal functions of the body need to be monitored. Patients ought to be told of possible side effects. Medication that is cost effective and produces benefits should be sought out ^20^ ^-^ ^21^.

The Brazilian Government provides some free medication for the treatment of neuropathic pain. They do not cost a lot but medium to high doses cannot taken due to the problems that can be caused as they are old medications. The side effects of the medication accounted for almost half (42.9%) of the patients terminating their treatment in this study.

The most effective medications such as duloxetine, pregabalin and gabapentine that have few side effects, are not provided for by the Government. This is due to their high costs. They are therefore out of reach for the majority of the patients with leprosy that have low incomes in Brazil ^8^ ^,^ ^22^.

7 patients (58.4%) out of the 12 who received medication for neuropathic pain did not experience improvements in their symptoms. The following were the causes identified for the failure in the treatment where the medication was used: side effects that caused the use of inadequate/insufficient doses of the medication, polytherapy not being used in cases where the therapeutic responses to a drug was unsatisfactory and an unawareness of the benefits of medication that are used for neuropathic pain. The following may also have contributed to the lack of improvements: a lack of close monitoring of the patients (follow-up checks having long intervals or changes in medical staff), unbelief in neuropathic pain diagnosis (they were given high doses of corticosteroids and analgesics associated with the medication for the neuropathic pain) and psychosocial causes.

The importance of treating neuropathic pain with medication is therefore clear, despite some of the difficulties. This is because the results showed significant improvements in the pain compared to those that were not treated. 

The subjectivity of the symptoms was the main limiting factor in this study. The multifaceted nature and intensity of the pain means that it cannot be objectively measured. This means that it can be under or over estimated. This can be seen as another obstacle that is related to the reduced number of patients with neuropathic pain (n=22). This limits other inferences that can be made based on the results of the study. The population that has been presented in this study, was treated as outpatients for one year in a specific region.

During the study, all of the patients that had not been diagnosed with neuropathic pain were given information about the illness. They were then given medication and were monitored by specialist in chronic pain.

## Conclusion

 We noted difficulties in diagnosing neuropathic pain for those with leprosy in that almost half of the patients that were studied had not had their pain diagnosed. One of the main reasons for this is because medical staff are not using, on a routine basis, an adequate protocol which would allow them to investigate and effectively diagnosis neuropathic pain. Complications and prolonged suffering is caused by misdiagnosis and inadequate treatment.

Neuropathic pain in patients that had been treated with leprosy reached 90.1% of cases. They stated that they had to deal with moderate and severe pain for more than six months. Therefore neuropathic pain for those with leprosy is an important cause for suffering.

The results show significant improvements in the symptoms for those that received treatment in comparison to those that did not. The reasons can be given for those that stated to be the same or who became worse after the therapy include: the high degree of side effects, insufficient doses of the medication used, and the non-use of polytherapy in cases in which the therapeutic responses to one drug was unsatisfactory.

We therefore conclude that treatment through the use of medication for neuropathic pain ought to be introduced, despite there not being many systematic and methodological studies in this area. This would go a long way in reducing human suffering.
